# *Physiological Reports* 10th anniversary year: Looking to the future

**DOI:** 10.14814/phy2.70043

**Published:** 2024-09-09

**Authors:** Josephine C. Adams

**Affiliations:** ^1^ School of Biochemistry University of Bristol Bristol UK

**Keywords:** physiology

## Abstract

*Physiological Reports* 10th anniversary year banner image.

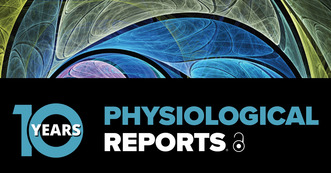

Over the last 9 months, *Physiological Reports* has featured special initiatives and journal content to mark its “10th Anniversary Year” of publication as a gold Open Access journal co‐owned by the American Physiological Society and The Physiological Society (Adams et al., 2023). Activities to date have included an expanded schedule of Calls for Papers (https://physoc.onlinelibrary.wiley.com/hub/journal/2051817x/call‐for‐papers) and the start‐up of Methods articles as a distinct article type. It is pleasing to see that authors are taking advantage of this offering, with articles already published on diverse topics including, for example, pancreatic islet isolation (Fernandes‐da‐Silva et al., 2024) or evaluation of ventricular–vascular coupling (Mulligan et al., 2024). Information on the specifications for submitting a Methods article can be found at (https://physoc.onlinelibrary.wiley.com/hub/journal/2051817x/about/author‐guidelines/methods_in_physiology).

The Editors have also inaugurated a “Paper of the Year” award to recognize the important research contributions of early career researchers (ECR). The eligible research articles (research papers with a single early career researcher as first author that had been published during the defined annual time period) were ranked in a two‐step process by the executive editors on the basis of sound science quality, the scientific contribution made and data presentation. From this process, we were delighted to make the first Paper of the Year award to Aural Leuchtmann, then a Ph.D. student in the laboratory of Dr. Christoph Handschin at University of Basel, Switzerland, and first author of the paper “Effects of high‐resistance wheel running on hallmarks of endurance and resistance training adaptations in mice” (Leuchtmann et al., 2023). This paper showed that two different exercise training modes had overlapping but differential outcomes for running performance, grip strength, and increased soleus muscle mass. An interview with Aural about the research and his career to date was subsequently published in The Physiological Society's *Physiology News* magazine (Physiology News Magazine, 2024).

During the first half of the anniversary year, *Physiological Reports* has published several perspectives on the first 10 years of the journal in the form of invited Editorials from the founding and former Editor‐in‐Chiefs and from current Editors of some of the APS and TPS research journals that support *Physiological Reports* through manuscript transfer. These Editorials can be found together online within an ongoing “10th Anniversary Collection” of publications (see https://physoc.onlinelibrary.wiley.com/doi/toc/10.1002/(ISSN)2051‐817X.10th‐Anniversary‐Collection).

In the final quarter of the anniversary year, *Physiological Reports* pivots to consider what the next decade of physiological research may bring. What areas of physiological research are expanding? Are there emerging new areas of high potential, perhaps driven by new technologies? What research directions will need to be prioritized to respond to new or increasing societal or environmental pressures? One ongoing special anniversary activity is the ECR competition to write a Short Review related to “The Future of Physiology,” for which the winners will be announced later this year. In addition, this issue sees the start of publication of an occasional series of invited Editorials, authored by representatives of national or international Physiological Societies from around the world. The goal is to share a diversity of perspectives on future needs and directions in physiological research. In this issue, we are pleased to publish an editorial by the President of the African Association of Physiological Sciences (AAPS; Essop, 2024), and an editorial by officers of the Australian Physiological Society (AuPS; Murphy et al., 2024). The editors of *Physiological Reports* hope that these updates and commentaries will be of general interest to the readership and may also provide a stimulus to facilitate discussions and networking or to inspire new researchers.

## FUNDING INFORMATION

No funding was associated with the preparation of this Editorial.

## ETHICS STATEMENT

The author is the Editor‐in‐Chief of Physiological Reports and was blinded from reviewing or making decisions for this manuscript. An alternate editor oversaw the manuscript process for this article.

## Data Availability

The data can be found within this article.
